# The Role of Targeted Osmotic Lysis in the Treatment of Advanced Carcinoma in Companion Animals: A Case Series

**DOI:** 10.1155/2022/2747108

**Published:** 2022-08-02

**Authors:** Harry J. Gould, Samantha Edenfield, Paige R. Miller, Kelly Jean Sherman, Brian Melius, Alissa Whitney, Robert P. Hunter, Fabio Del Piero, Dennis Tracey, Dennis Paul

**Affiliations:** ^1^Department of Neurology, Louisiana State University Health Sciences Center, New Orleans, LA, USA; ^2^Oleander Medical Technologies, Baton Rouge and New Orleans, LA, USA; ^3^Department of Pharmacology and Experimental Therapeutics, USA; ^4^Metairie Small Animal Hospital, Metairie, LA, USA; ^5^Monroe Street Animal Hospital, Mandeville, LA, USA; ^6^One Medicine Consulting, Olathe, KS, USA; ^7^Department of Pathobiological Sciences and Louisiana Animal Disease Diagnostic Laboratory (LADDL), Louisiana State University School of Veterinary Medicine, Baton Rouge, LA, USA; ^8^Big Easy Medical, 6818 Memphis St New Orleans, LA 70124, USA

## Abstract

**Background:**

Targeted osmotic lysis (TOL) is a novel technology that involves concomitant stimulation of voltage-gated sodium channels (VGSCs) and the pharmacological blockade of Na^+^, K^+^-ATPase causing lysis of highly malignant cancer cells. *Hypothesis/Objectives*. TOL offers an option for treating advanced carcinomas in companion animals. *Animals*. Two cats and 2 dogs that presented to veterinary hospitals for evaluation and treatment of one of several forms of carcinoma.

**Methods:**

Digoxin was administered to achieve steady-state, therapeutic concentrations. The animals were then exposed to pulsed electric field stimulation. Pre- and posttreatment assessments of tumor size and quality of life were compared. The treatment frequency and survivability varied, based on the patient's premorbid functioning and response to treatment.

**Results:**

Regardless of cancer type, TOL consistently increased survival beyond expected, often improving, but without compromising of quality of life. *Conclusions and Clinical Importance*. TOL warrants consideration as an option for managing advanced carcinomas.

## 1. Introduction

Fifty percent of dogs and 32% of cats over the age of 10 will be diagnosed with cancer in their lifetime. [1] Although surgery, radiation, and chemotherapy have been shown to prolong life, these treatment modalities can be toxic, physically taxing, and have the potential to reduce the quality of life. TOL is a novel technology that produces lysis of highly malignant cancer cells by simultaneously activating VGSCs and pharmacologically blocking Na^+^, K^+^-ATPase with a cardiac glycoside. [2, 3] Because the cells of highly malignant carcinomas in humans and mice overexpress VGSCs at levels many times greater than even normal excitable cells, [2–7] we hypothesized that because of the conserved nature of the VGSC/Na^+^, K^+^-ATPase mechanism, the same would be true in companion animals.

## 2. Materials and Methods

### 2.1. Drugs

Digoxin was administered orally by owners: cats, 0.0035 mg/kg every other day and dogs, 0.009 mg/kg twice daily.

### 2.2. Animals

Each animal considered for TOL treatment had advanced carcinoma that was generally refractory to chemo- and/or radiotherapy. All treatments were conducted according to a protocol approved by the LSU Health Sciences Center Institutional Animal Care and Use Committee.

### 2.3. Pulsed Electric Field Stimulation

**Figure 1:**
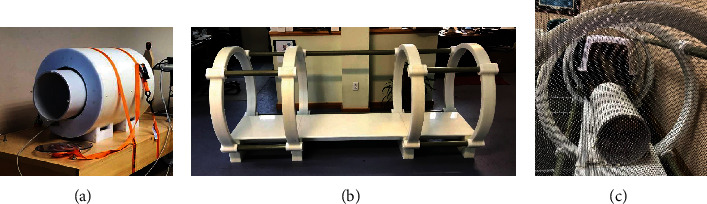
The photograph in (a) depicts the toroidal device used to deliver the uniform PEFs with to open VGSCs; maximum field amplitude of 6 V/m. The photograph in (b) depicts the coaxial ring device used to deliver PEFs with a field amplitude of 18 V/m. (c) Dogs in the carriers that allowed room for *ad libitum* movement to adjust position for comfort during the period of stimulation while being treated with TOL.

Each round of TOL consisted of Na^+^, K^+^-ATPase blockade, and PEF stimulation administered on 2 consecutive days. Digoxin was administered for 4 dosing cycles, and an additional dose was administered prior to each period of stimulation to establish and maintain steady-state concentrations during treatment. The patients were then placed in the bore of a toroidal or a coaxial ring stimulating device (Figure 1; The Phantom Laboratory, Salem, NY) that delivered a uniform PEF known to effectively activate VGSCs (6 V/m field amplitude in the toroid device for 3 hours or 18 V/m in the coaxial ring device for 2 hours, delivered as a 10 msec positive/negative square wave pulse and a 15 msec interstimulus interval). [8] The patients were monitored by the attending veterinarian and the owner for signs of discomfort and adverse cognitive or behavioral effects during and after stimulation and were then sent home, to return the next day for an additional dose of digoxin and the second round of stimulation.

### 2.4. Image Analysis

Biopsies of each cancer were obtained, fixed, and immunohistochemically stained for VGSC expression with an antibody that recognized a conserved segment of the channel. Images were taken on a Leica TS SP8 confocal microscope and examined for histofluroescent labeling. Histopathological analysis of tumor samples stained with hematoxylin and eosin was performed by a board-certified veterinary pathologist who was blinded to the treatments provided. CT scans or X-ray images were obtained and reviewed and cross-sectional dimensions were measured before and at intervals after treatment.

## 3. Case Histories

### 3.1. Case 1: Robert—6-Year-Old Male Cat with Poorly Differentiated Nasopharyngeal Adenocarcinoma

**Figure 2:**
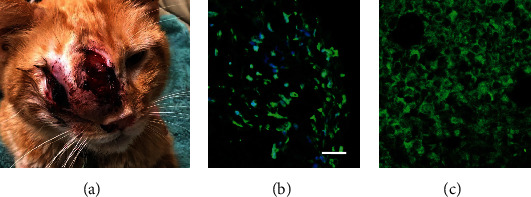
The photograph in (a) depicts the appearance of a large mass that involved the right orbit and at the time of presentation had erupted through the skin as an open wound over the bridge of the nose and face. The photomicrograph in (b) depicts the prominent immunohistofluorescent labeling of VGSCs (green) observed in a tissue sample taken from the tumor in (a) before treatment with TOL. For comparison, the photomicrograph in (c) depicts a similarly processed tissue sample showing VGSC expression in a murine homograft that was assessed in a pre-clinical study to determine the efficacy of TOL in treating triple-negative breast cancer. The calibration bar for photomicrographs in *B* = 25 *μ*m and nuclei (blue) are labeled with DRAQ5.

**Figure 3:**
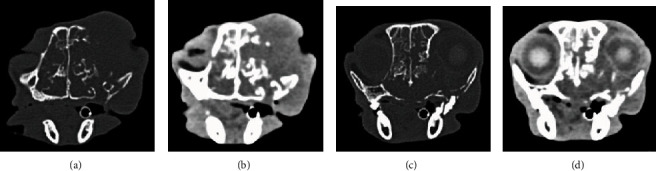
The CT images depict soft tissue (b, d) and bone window (a, c) images of the rostral (a, b) and caudal (c, d) portions of the tumor shown in Figure 1 prior to treatment with TOL. Note that the tumor involves and extends beyond the margins of the right orbit, invades the paranasal sinuses bilaterally, and perforates the hard palate extending into the oral pharynx.

The patient presented with a 10-month history of intermittent dysphagia, rhinitis and epistaxis, epiphora, nasal swelling, and weight loss and displayed a prominent mass that had erupted through the skin as an open wound over the bridge of the nose and face and involved the right orbit (Figure 2(a)). A CT scan revealed a large tumor that occupied the right nasal cavity, had invaded the orbit and nasal turbinate bones, and extended inferiorly to involve the hard palate (Figure 3). Due to the lack of response to standard chemotherapy and continued deterioration in the quality of life, humane euthanasia was scheduled 3 days hence. Based upon a review of preclinical evidence in mice and the favorable results of safety trials performed in 2 normal dogs and 2 normal cats, the treating veterinarian suggested a treatment trial with TOL (unpublished data).

A tissue biopsy was obtained that revealed the level of VGSC expression was consistent with that observed prior to treatment in mice and was considered sufficiently high to anticipate a favorable response to treatment with TOL (Figures 2(b) and 2(c)).

**Figure 4:**
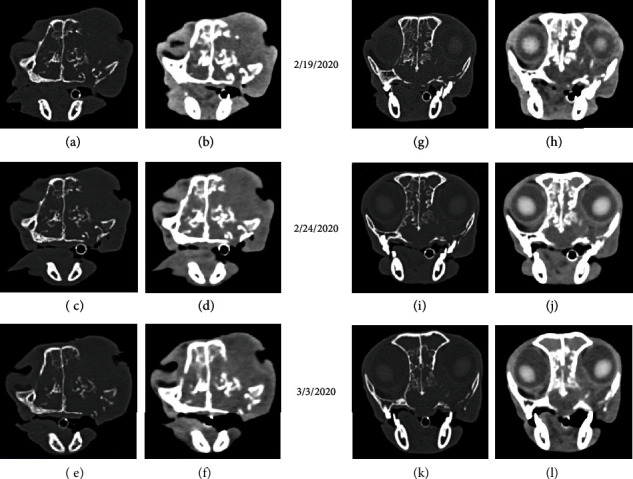
The CT images depict soft tissue and bone window images of closely comparable rostral (a-f) and caudal (g-l) portions of the tumor shown before (a, b, g, h) and after (c, d, i, j, e, f, k l) each of 2 rounds of treatment with TOL. Despite the improvement in the patient's level of social interaction and appetite, there was no evidence to support a reduction in the tumor mass.

**Figure 5:**
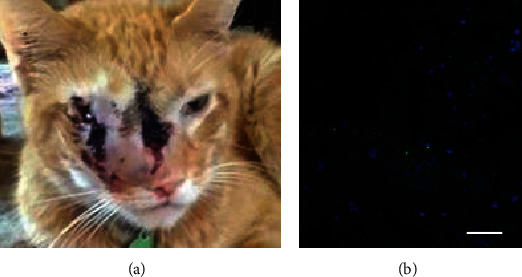
The photograph in (a) depicts the appearance of the mass presented Figure 1 after 2 rounds of treatment with TOL. Note the reduction in localized edema revealing more of the globe of the eye. The photomicrograph in (b), compared to the photomicrograph in Figure 1(b), depicts the relative absence of cells that highly express VGSCs (green) following treatment with TOL. The calibration bar = 25 *μ*m.

The patient spent the majority of the time during the first day of stimulation sleeping and showed no signs of discomfort or adverse cognitive or behavioral effects during or after treatment. The 2^nd^ day was uneventful, with the exception that large amounts of necrotic tissue were expelled from the center of the defect in the skin, and frequent sneezes ejected small pieces of tissue from the nose and oral cavity. CT imaging obtained 1 week after treatment indicated that the size of the tumor had not changed (Figure 4). By contrast, the area of tumor visible on the surface had decreased sufficiently to allow more of the orbit to be discernible (Figure 5(a)). A posttreatment biopsy revealed very few cells that highly expressed VGSCs (Figure 5(b)).

Two additional rounds of treatment were administered over the next 2 weeks. It was noted that the main visible tumor mass continued to decrease in size and that the patient's level of social interaction and appetite improved, but the morning after the third round of treatment, the patient showed no interest in eating. Examination of the oral cavity revealed a patent defect in the hard palate and the oral portion of the tumor was gone. Although the cat's behavior seemed to improve over the next 24 hours, his ability to eat and overall condition did not. Euthanasia was conducted 8 days after his last treatment with TOL; 29 days after presentation to clinic.

### 3.2. Case 2: Gizmo—11-Year-Old Male Maltese Mix with Facial Adenocarcinoma

**Figure 6:**
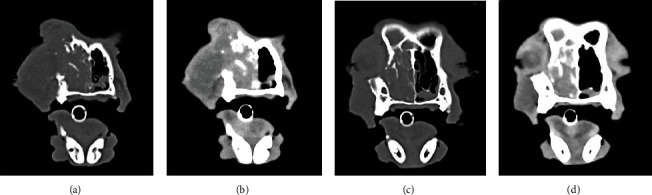
The CT images depict soft tissue (b, d) and bone window (a, c) images of the rostral (a, b) and caudal (c, d) portions of a large facial carcinoma that involved the right orbital wall and nasal turbinate bones and had extended into the paranasal sinuses.

**Figure 7:**
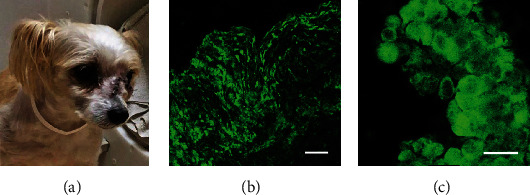
The photograph in (a) depicts the appearance of a large mass that involved the right orbit and at the time of presentation had erupted through the skin over the bridge of the nose and face. The low-power (b) and high-power (c) photomicrographs depict the prominent immunohistofluorescent labeling of VGSCs (green) revealed in a tissue sample taken from the tumor in (a) before treatment with TOL. The calibration bar for photomicrographs in (b) and (c) is 50 and 25 *μ*m, respectively.

The patient was referred for treatment of a large facial carcinoma that involved the left orbital wall and nasal turbinate bones and had extended into the paranasal sinuses (Figures 6 and 7(a)). The tumor was not responding to chemotherapy and survival was anticipated to be 3 to 6 months. VGSC expression revealed in a tissue biopsy indicated the likelihood of a favorable response to treatment with TOL (Figures 7(b) and 7(c)).

**Figure 8:**
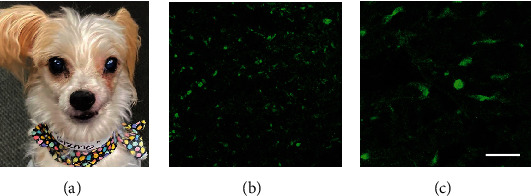
The photograph in (a) depicts the appearance of the mass presented in Figure 7 after debulking, cauterization and 4 rounds of treatment with TOL. The photomicrograph in (b), compared to the photomicrograph in Figure 7(b) and Figure 7(c), depicts the significant reduction in the number of cells that highly express VGSCs (green) following treatment with TOL. The calibration bar in (c) is 25 *μ*m.

Over the next 10 weeks, the patient received 5 rounds of treatment with TOL. The first 4 rounds of PEF stimulation were administered in the toroidal device that delivered a maximum field amplitude of 6 V/m. No signs of discomfort or signs of adverse cognitive or behavioral effects were observed during the 3-hour periods of stimulation or after treatment. Posttreatment physical examination revealed that the rostral portions of the mass appeared less infiltrative and were more encapsulated, but caudally, at the level of the maxilla and orbit, the tumor seemed to have invaded the bone more extensively. Posttreatment biopsy revealed a significant reduction in the number of cells that highly expressed VGSCs (Figures 8(b) and 8(c)). Two weeks later, a fifth and final round in the initial series of treatments with TOL was administered using a newly designed coaxial ring device (Figures 1(b) and 1(c)) that made it possible to deliver a uniform PEF amplitude of 18 V/m. At this field amplitude, a total of 2 hours of stimulation was determined to provide maximum efficacy in mice (unpublished results) and the stimulus parameters were adjusted accordingly. No adverse reactions were observed with the increase in field strength. Despite the owner's report that the patient appeared to be “feeling great” and back to normal and that the patient continued to be active, have a normal appetite, and neither lost nor gained weight, CT imaging indicated that the treatments were having little effect on overall tumor size. Treatment with TOL was discontinued and replaced with a new chemotherapeutic regimen.

**Figure 9:**
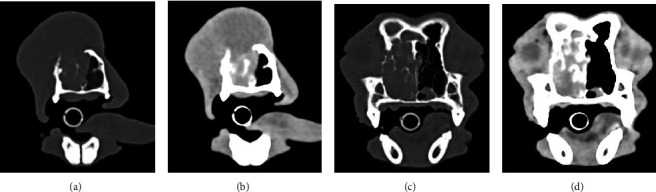
The soft tissue (b, d) and bone window (a, c) CT images obtained approximately 6 months after the images presented in Figure 6 approximate the levels shown in Figure 6. Despite palliative debulking procedures and 6 months of chemotherapy, no appreciable changes were noted in the deep-seated portions of the tumor.

Over the next 5 months, the patient generally did well. The size of the rostral portion of the tumor had decreased and was no longer visible on gross examination (Figure 8(a)), but the caudal portion of the tumor continued to grow and destroy more of the facial bones. Despite palliative debulking procedures, a CT scan obtained after the 6 months of chemotherapy revealed that there had been no appreciable change in the size of the deep-seated portions of the tumor (Figure 9). Because of the minimal progress, it was decided to restart treatment with TOL. The patient received an additional 3 rounds of TOL but began experiencing frequent seizures, and imaging revealed greater damage to the facial bones. It was concluded that it would be unlikely that additional treatments with TOL would be beneficial or improve prognosis and may in fact be detrimental if the tumor were eliminated or significantly reduced, as the mass was now providing much of the support for the facial tissues. Euthanasia was performed due to seizures and probable metastasis to the brain, 11 months after the first TOL treatment.

### 3.3. Case 3: Dodge—15-Year-Old Male English Shepherd with Bronchoalveolar Adenocarcinoma

**Figure 10:**
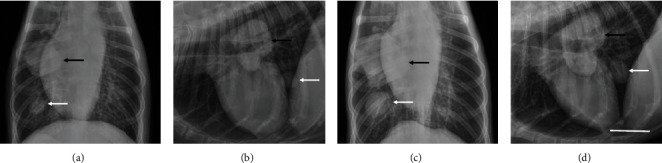
The chest X-rays in (a) and (b) depict the AP and RL appearance of 2 masses in the right lung prior to treatment with TOL. The larger mass (black arrows) is located centrally in the middle lobe in close proximity to the hilus of the lung and smaller mass (white arrows) is located more caudal in the cranial lobe. (c, d) The appearance of the same masses 7 months later after receiving 7 rounds of treatment with TOL. Taking account of the positioning in the X-ray machine, the size of the tumors varied but appeared to have increased gradually by approximately 28% over the 7 1/2 month course of treatment. The calibration bar in (d) is 2.5 *μ*m.

The patient was referred for evaluation and treatment of 2 masses that had been discovered incidentally in the right lung (Figure 10). At presentation, the patient showed signs of mild to moderate cognitive impairment and significant bilateral osteoarthritis in the hips that restricted range of motion and his ability to ambulate. Because the prognosis for this type of cancer with evidence of metastases is typically 8-12 months from the time of diagnosis and the carcinoma was considered unresectable, a treatment trial with TOL was suggested even though no standard treatment modalities had been tried.

**Figure 11:**
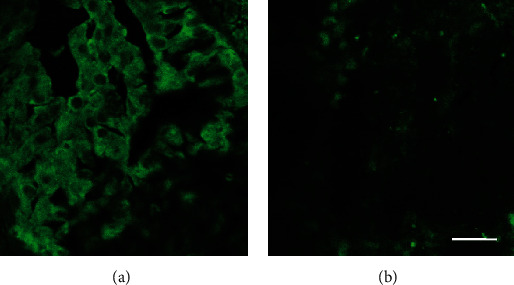
The photomicrograph in (a) depicts the immunohistofluorescent labeling of VGSCs (green) observed in a tissue sample taken from the larger tumor mass before treatment with TOL. The immunohistofluorescent evaluation of a tissue sample obtained after treatment with TOL in B reveals the significant reduction in the number of cells that highly express VGSCs. The calibration bar in (b) is 25 *μ*m.

Assessment of a true-cut biopsy identified the tumor as a bronchoalveolar adenocarcinoma and that the level of VGSC expression supported treatment with TOL (Figure 11(a)). Three weeks after pretreatment X-rays were obtained, treatment with TOL was initiated and administered without signs of adverse cognitive or behavioral effects. A set of posttreatment X-ray images were obtained 4 weeks after the first round of treatment revealed an approximate 15% increase in tumor size. Despite the increased size, the lack of treatment-associated adverse effects was deemed sufficient to request a second round of TOL. Again, posttreatment X-rays revealed a small increase in tumor size. In the lack of adverse effects to compromise the quality of life, the treatment was again viewed favorably and it was deemed reasonable to continue regular treatments with TOL. Despite stable appetite and behavior and the absence of adverse pulmonary symptoms, e.g., dyspnea, cyanosis, or hypoxia, the owners decided that it was time to euthanize the patient because of his poor quality of life associated with severe osteoarthritis and progressive dementia.

Overall the patient received treatment every 3-4 weeks for a total of 8 rounds of treatment over the course of 33 weeks. The final X-ray, obtained 14 days before his death, showed that the tumor measured approximately 28% larger than the initial measurements but had decreased by approximately 14% compared to the images obtained prior to the last treatment, indicating that tumor growth had been halted or drastically slowed over the course of treatment.

**Figure 12:**
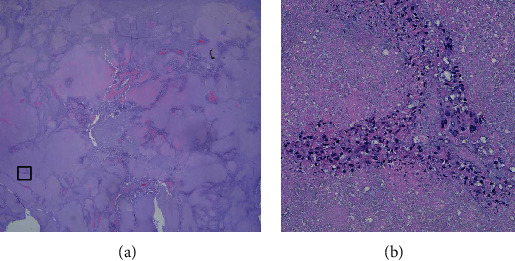
The photomicrograph in (a) is a low-power (X1) image of a representative portion of the lung tumor that had received 8 rounds of treatment with TOL alone over 7 1/2 months. The tissue was taken at the time of necropsy and stained with hematoxylin and eosin. Note the large areas of necrosis that comprise over 90% of the tumor mass. The photomicrograph in (b) is a X40 enlargement of the tissue field defined by the box in (a) that illustrates in greater detail the few blood vessels and neoplastic cells that remained.

Euthanasia was performed 7.5 months after the initial treatment with TOL, and a necropsy was conducted. Gross and histopathologic analysis determined that necrosis, located both centrally and in portions of the tumor closer to its outer margins, comprised greater than 90% of the soft, white tumor (Figure 12(a)). Only a few blood vessels with adjacent neoplastic cells remained (Figure 12(b)). There was no evidence of infection or other intrinsic, predisposing factor of necrosis. An additional sample was collected for immunohistochemical evaluation that revealed that most of the cells that highly expressed VGSCs were no longer present (Figure 12(b)), consistent with the interpretation that TOL had and was continuing to destroy the cells that had the highest levels of VGSC expression.

### 3.4. Case 4: Baby Cat—6-Year-Old Female Savannah-F2 Cat with Oral Squamous Cell Carcinoma

**Figure 13:**
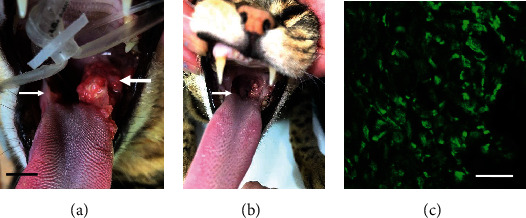
The photographs in (a) depicts the appearance of a large tumor located in the left oral pharynx (large arrow) and a second, smaller mass on the right (small arrow) that was discovered on initial physical examination (a). The photograph in (b) depicts the tumors midway through a third treatment with TOL. Note that the smaller tumor is no longer apparent (small arrow) and the larger tumor is significantly reduced. The calibration bar in (a) is 3 mm. The photomicrograph in (c) depicts the immunohistofluorescent labeling of VGSCs observed in a tissue sample taken from the tumor in (a) before treatment with TOL. Note that TOL was the only treatment provided in the management of the tumors. The calibration bar in (c) is 25 *μ*m.

The patient presented for evaluation excessive sialorrhea and licking. Examination revealed the presence of prominent oral mass occupying the left posterior lateral oral pharynx involving the soft palate, the upper and lower molar teeth, and extending anteriorly along the lateral margin of the tongue (Figure 13(a)). A second, smaller mass was also noted in the oral pharynx on the right. A fine needle aspirate identified the mass as a probable squamous cell carcinoma. Based on gross appearance of the tumor and past clinical experience, life expectancy was anticipated to approximate 30-60 days regardless of treatment. [9, 10] Upon consideration of the results achieved in the previous cases, a biopsy was obtained that revealed VGSC expression sufficiently high to anticipate a favorable response to treatment with TOL (Figure 13(c)) and a trial treatment was arranged.

The cat received 3 rounds of treatment 3 weeks apart without discomfort or adverse effects. The sialorrhea resolved after the first treatment. Examination of the oral cavity after the first day of the third round of TOL revealed that the main tumor mass was much smaller and appeared “much less angry” than when first observed (Figure 13(b)) and the smaller tumor mass was no longer apparent. The cat has completed 6 rounds of treatment and continues to do well 5 months after the tumor was discovered. Because of the cat's positive response to treatment, the current plan is to continue TOL treatment monthly until the tumor has resolved or the disease is stable.

## 4. Discussion

We have presented observations on the efficacy and tolerability of TOL, a new and fundamentally different approach to treating cancer (supplementary file 1), that may have a role in the management of advanced disease in companion animals. Despite the lack of tumor resolution, treatment with TOL, (1) consistently increased survival beyond that expected at the time of diagnosis, (2) was well-tolerated without apparent adverse effects, and (3) was associated with the extension of life without compromising and in some instances improving quality of life. [3] Although we were generally unable to eliminate the tumor masses, size reduction was noted in 2 of the 4 cases. In most cases, tumor growth seemed to stabilize or slow and in 1 case, the residual mass was predominantly necrotic. Humane termination of life was due to the progression of comorbid conditions that compromised essential organ function or quality of life rather than cancer progression.

The evidence that the therapeutic effects of TOL are similar and consistent regardless of species or cancer type supports our original hypothesis that because the VGSC/Na^+^, K^+^-ATPase relationship is a conserved mechanism for communication and survival in the animal kingdom and basic to survival, the therapeutic benefits of TOL should apply to all forms of cancer that overexpress this basic mechanism thus offering a safe, well-tolerated and broadly effective treatment option that can reduce or stabilize tumor growth and increase survival of patients with advanced carcinomas without compromising quality of life. The clear exception to this observation is in instances where a cancer by virtue of invading normal supportive elements or organ integrity compromises structural integrity and by default replaces the defects with less acceptable reinforcing tissue that is susceptible to TOL and subject to elimination. Such defects should contraindicate treatment or be addressed prior to initiating TOL.

In conclusion, we propose that TOL's role and positioning in the therapeutic algorithm of care in veterinary practice warrants further study. Alone, TOL may provide a means to reduce or stabilize a tumor prior to resection or to eliminate remnants of disease after removal. From the current study, it would appear from the current series that TOL may also serve as an adjunct to chemotherapy and potentially other forms of targeted therapy for a variety of advanced carcinoma.

## Data Availability

Data available on request from the corresponding author.

## Abbreviations

AP: Anterior-posterior
CT: Computerized tomographic
EOD: Every other day
LL: Left lateral
MSAH: Metairie Small Animal Hospital
MWF: Monday/Wednesday/Friday
Na^+^, K^+^-ATPase: Sodium, potassium-ATPase
PBS: Phosphate-buffered saline
PEF: Pulsed electric field
RL: Right lateral
SID: Once daily
TLR7: Toll-like receptor 7
TOL: Targeted osmotic lysis
T/T/S/S: Tuesday/Thursday/Saturday/Sunday
VGSCs: Voltage-gated sodium channels.

## Additional Points

*Patents*. A patent for the technology described in this manuscript entitled, Targeted Osmotic Lysis of Cancer Cells—file no. 11M01 (serial no. 13/552,909) Paul DJ and Gould HJ III was allowed on 12/30/2014.

## Supplementary Materials

Supplementary Materials File 1 provides the rationale and an explanation for the basic process involved in targeted osmotic lysis.
