# Supplementary material for: The Role of Targeted Osmotic Lysis in the Treatment of Advanced Carcinoma in Companion Animals: A Case Series
Source: Case Rep Vet Med. 2022 Aug 2;2022:2747108. doi: 10.1155/2022/2747108 (PMC9363929; doi:10.1155/2022/2747108)
Supplement: Supplementary Materials — File 1 provides the rationale and an explanation for the basic process involved in targeted osmotic lysis. [file 2747108.f1.docx]

**The Role of Targeted Osmotic Lysis in the Treatment of Advanced Carcinoma in Companion Animals, A Case Series**

**A novel approach to cancer therapy.** TOL is a novel therapeutic modality that recognizes that basic biological mechanisms essential for cell function and survival may be subject to manipulation that can affect the treatment of disease in a fundamentally different way than the methods in current use. TOL is based on the observation that many cancers, especially in late-stage, over-express VGSCs and Na^+^, K^+^-ATPase, ^1-4^ thereby increasing their ability to invade normal tissue and to metastasize. ^5-10^ TOL takes advantage of the VGSC/Na^+^, K^+^-ATPase relationship that is essential for cell survival because of its role in the maintenance of membrane potentials and cellular homeostasis. Unlike many treatment methods ^5-10^ that target the VGSCs for destruction or impairing function, TOL enhances VGSC activity thereby greatly increasing sodium influx while simultaneously blocking the sodium pumping mechanism thereby preventing the extrusion of these ions. ^20,21^ The osmotic influx of water floods the cells beyond their capacity to comply, resulting in lysis of the cells. ^22^ Normal cells that require and express far fewer VGSCs than advanced neoplastic cells conduct significantly less sodium and absorb less water. Consequently, they are spared from permanent effects of the TOL process, ^22^ thus providing a very selective treatment affecting only those cells that greatly over-express VGSC without imposing significant morbidity due to destruction of VGSCs that are also essential for the survival and function of normal cells. Improvements in survival following TOL therapy appears to be at least comparable to that reported in response to treatment of grade III/stage III bronchoalveolar adenocarcinoma with chaperone-rich cell lysate vaccine and imiquimod, a topical TLR7 agonist ^23^ and may serve as an option in cases of disease resistant to standard chemotherapy.

**REFERENCES**

1. Onkal R, Djamgoz MB. Molecular pharmacology of voltage-gated sodium channel expression in metastatic disease: Clinical potential of neonatal NaV1.5 in breast cancer. *Eur J Pharmacol* 2009;625:206-219.
2. Djamgoz MB, Onkal R. Persistent current blockers of voltage-gated sodium channels: A clinical opportunity for controlling metastatic disease. *Recent Patents on Anti-cancer Drug Discovery* 2013;8:66-84.
3. Fraser SP, Diss JKJ, Chioni AM, et al. Voltage-gated sodium channel expression and potentiation of human breast cancer metastasis. *Clin Cancer Res* 2005;11:5381–5389.
4. Fraser SP, Ozerlat-Gunduz I, Brackenbury WJ, et al. Regulation of voltage-gated sodium channel expression in cancer: hormones, growth factors and auto-regulation. *Philos Trans R Soc Lond B Biol Sci* 2014;369:20130105.
5. Djamgoz MBA, Mycielska M, Madeia Z, et al. Directional movement of rat prostate cancer cells in direct-current electric field: involvement of voltage-gated Na+ channel activity. *J Cell Sci* 2001;114:2697-2705.
6. Bennett ES, Smith BA, Harper JM. Voltage-gated Na+ channels confer invasive properties on human prostate cancer cells. *Pfugers Arch* 2004;447:908-914.
7. Fiske JL, Fomin VP, Brown ML, et al. Voltage-sensitive ion channels and cancer. *Cancer Metastasis Rev* 2006;25:493-500.
8. Brackenbury WJ, Chioni AM, Diss JKJ, et al. The neonatal splice variant of Nav1.5 potentiates in vitro invasive behaviour of MDA-MB-231 human breast cancer cells. *Breast Cancer Res Treat* 2007;101:149-160.
9. Roger S, Rollin J, Barascu A, et al. Voltage-gated sodium channels potentiate the invasive capacities of human non-small-cell lung cancer cell lines. *Int J Biochem Cell Biol* 2007;39:774-786.
10. Roger S, [Potier M](http://0-www.ncbi.nlm.nih.gov.innopac.lsuhsc.edu/pubmed?term=%22Potier%20M%22%5BAuthor%5D), [Vandier C](http://0-www.ncbi.nlm.nih.gov.innopac.lsuhsc.edu/pubmed?term=%22Vandier%20C%22%5BAuthor%5D), et al. Voltage-gated sodium channels: new targets in cancer therapy? *Curr Pharm Des* 2006;12:3681-3695.
11. Brackenbury WJ, [Isom LL](http://0-www.ncbi.nlm.nih.gov.innopac.lsuhsc.edu/pubmed?term=%22Isom%20LL%22%5BAuthor%5D) Voltage-gated Na+ channels: potential for beta subunits as therapeutic targets. *Expert Opin Ther Targets* 2008;12:1191-1203.
12. Wuethrich PY, Schmitz S-FH, Kessler TM, et al. Potential influence of the anesthetic technique used during open radical prostatectomy on prostate cancer-related outcome: a retrospective study. *Anesthesiology* 2010;113:570-576.
13. Mao L, Lin S, Lin J. The effects of anesthetics on tumor progression. *Int J Physiol Pathophysiol Pharmacol* 2013;5:1-10.
14. [Leslie TK](https://www-ncbi-nlm-nih-gov.ezproxy.lsuhsc.edu/pubmed/?term=Leslie%20TK%5BAuthor%5D&cauthor=true&cauthor_uid=31348974), [James AD](https://www-ncbi-nlm-nih-gov.ezproxy.lsuhsc.edu/pubmed/?term=James%20AD%5BAuthor%5D&cauthor=true&cauthor_uid=31348974), [Zaccagna F](https://www-ncbi-nlm-nih-gov.ezproxy.lsuhsc.edu/pubmed/?term=Zaccagna%20F%5BAuthor%5D&cauthor=true&cauthor_uid=31348974), et al. Sodium homeostasis in the tumour microenvironment. [*Biochim Biophys Acta Rev Cancer*](https://www-ncbi-nlm-nih-gov.ezproxy.lsuhsc.edu/pubmed/?term=leslie+tk+james+ad) 2019;1872:188304.
15. Exadaktylos AK, Buggy DJ, Moriarty DC, et al. Can anesthetic technique for primary breast cancer surgery affect recurrence or metastasis? *Anesthesiolog* 2006;105:660-664.
16. Driffort V, Gillet L, Bon E, et al. Ranolazine inhibits NaV1.5-mediated breast cancer cell invasiveness and lung colonization. *Mol Cancer* 2014;13:264.
17. Nelson M, Yang M, Dowle AA, et al. The sodium channel-blocking antiepileptic drug phenytoin inhibits breast tumour growth and metastasis. *Mol Cancer* 2015;14:13.
18. Martin F, Ufodiama C, Watt I, et al. Therapeutic value of voltage-gated sodium channel inhibitors in breast, colorectal and prostate cancer: a systemic review. *Front Pharmacol* 2015;6:273.
19. Dutta S, Charcas OL, Tanner S, et al. Discovery and evaluation of nNa1.5 sodium channel blockers with potent cell envasion inhibitory activity in breast cancer cells. *Bioorg Med Chem* 2018;26:2428-2436.
20. Gould HJ 3^rd^, Norleans J, Ward TD, et al. Selective lysis of breast carcinomas by simultaneous stimulation of sodium channels and blockade of sodium pumps. *Oncotarget* 2018;9:15606-15615.
21. Paul D, Maggi P, Piero FD, et al. Targeted osmotic lysis of highly invasive breast carcinomas using a pulsed magnetic field and pharmacological blockade of voltage-gated sodium channels. *Cancers* 2020;12:1420-1433.
22. Paul D, Soignier RD, Minor L, et al. Regulation and pharmacological blockade of sodium-potassium ATPase: Inflammation may lead to neuropathy. *J Neurol Sci* 2014;340:139-143.
23. Epple LM, [Bemis](https://pubmed-ncbi-nlm-nih-gov.ezproxy.lsuhsc.edu/?term=Bemis+LT&cauthor_id=23786302) LT, [Cavanaugh](https://pubmed-ncbi-nlm-nih-gov.ezproxy.lsuhsc.edu/?term=Cavanaugh+RP&cauthor_id=23786302) RP, et al. Prolonged remission of advanced bronchoalveolar adenocarcinoma in a dog treated with autologous, tumour-derived chaperone-rich cell lysate (CRCL) vaccine. *Int J Hyperthermia* 2013;29:390-398.
